# Resistant or Susceptible? How Central European Oat (*A. sativa* L.) Cultivars React to *B. graminis* f. sp. *avenae* Infection

**DOI:** 10.3390/plants12223825

**Published:** 2023-11-11

**Authors:** Magdalena Cieplak, Sylwia Okoń

**Affiliations:** Institiute of Plant Genetics, Breeding and Biotechnology, University of Life Science in Lublin, 20-950 Lublin, Poland; magdalena.cieplak@up.lublin.pl

**Keywords:** *Avena sativa* L. cultivars, powdery mildew, resistance

## Abstract

In accordance with the postulates of integrated plant protection, the use of cultivars with genetically determined resistance is one of the main strategies for preventing losses caused by fungal pathogens. The development of breeding programs aimed at increasing resistance to pathogens should be preceded by a characterization of the resistance of cultivars grown in a given area. This allows us to determine the number of genes used in breeding and their effectiveness. It also allows us to estimate the pressure that the pathogen may exert on varieties with specific resistance genes. The presented work aimed to determine the level of resistance of oat varieties currently cultivated in Central Europe and the number of effective powdery mildew resistance genes currently used in oat breeding programs. The research showed that out of 46 varieties, only 5 were resistant to powdery mildew. Analysis of the infection profiles allowed us to postulate the presence of the *Pm7* gene in four of them. In the Merlin variety from the Czech Republic, it was not possible to determine which of the previously described genes determines resistance to powdery mildew. Due to the observed climate changes and the rapid adaptation of pathogens to new environmental conditions, it is crucial to introduce a wider pool of genes that determine the pathogen resistance of cultivars.

## 1. Introduction

The common oat (*Avena sativa* L.) is a cereal that originated from the Mediterranean region and is widely used in agroeconomy. It is used as human food and livestock fodder, either in the form of green forage or silage, particularly for poultry and horses. Additionally, this type of grain is used as a winter ground cover with zero crop rotation [1,2,3,4]. In most European cereal-growing areas, the major airborne diseases affecting oats and other cereals include rusts like leaf rust, brown rust, crown rust, Fusarium head blight, and powdery mildew [5,6,7]. The protection of cereals against fungal pathogens is a complex process combining biological, agrotechnical, and chemical methods. According to the postulates of integrated disease management, the priority in the fight against fungal diseases should be the introduction of forms with genetically determined resistance. In oats, one of the most harmful diseases is powdery mildew caused by *Blumeria graminis* f. sp. *avenae* Em. Marchal. These fungi significantly reduce the yield and quality of grain and forage. Oat powdery mildew is common in many parts of the world, such as Northwestern, Central, and Eastern Europe, and South and North America [8,9]. It occurs in Europe in countries such as Poland, Austria, the Czech Republic, Germany, and Greece [9,10]. Recently, there have also been outbreaks of this disease in some regions of China and the North-western Himalayas [11,12]. Crop losses caused by this disease in warm and humid years favorable for the development of these fungi are significant, and they range from 5 to 10% to 39% [8,13,14]. These fungi have effective dispersal mechanisms that make them difficult to control using crop management methods such as crop rotation. Oat powdery mildew can be controlled by fungicides; however, in many countries, the range of fungicides approved for oats is limited compared to other cereal species. Therefore, the most effective, economical, and environmentally friendly method of controlling these diseases in oats is breeding resistant cultivars [15,16]. The relationship between the host and powdery mildew is closely related to the “gene-for-gene” hypothesis, which says that the avirulence gene (*Avr*) in the pathogen’s genome is directed against a resistance (*R*) gene in the plant [17,18]. To date, 12 major genes conferring powdery mildew resistance in oats have been identified as *Pm1*–*Pm12* [19,20,21,22]. Breeding crops for disease resistance is crucial as attacks from pathogens can significantly reduce crop yield and grain quality in susceptible cultivars. However, there is little information available on the level of resistance of common oat cultivars to powdery mildew [23,24,25,26,27,28]. Knowing the level of resistance of currently cultivated varieties is the basis for building effective breeding programs; therefore, it is important to determine the resistance genes present in cultivated forms [29]. This study aims to determine the resistance of cultivars currently grown in Central Europe (Poland, the Czech Republic, and Germany) to the pathogen causing powdery mildew. The results obtained from this work will provide basic information that can help estimate the pressure that varieties with resistance genes may exert on the pathogen’s population to overcome this resistance.

## 2. Results

To determine the resistance level of oat cultivars to powdery mildew, 10 specific single-spore isolates of *B. graminis* f. sp. *avenae* were selected. These isolates were characterized based on their different levels of virulence against control lines and cultivars that are known to have powdery mildew resistance genes (Table 1).

By studying the infection profiles of these isolates, we were able to differentiate all known powdery mildew resistance genes in oats. Specifically, we observed a complete absence of infection symptoms in the Cc37722, AV1860, and Am25 genotypes, which carried the *Pm2*, *Pm4*, and *Pm5* genes, respectively. The cultivars ‘Jumbo’, ‘Mostyn’, and ‘Bruno’ have the *Pm1*, *Pm3*, and *Pm6* genes, but they were infected by most isolates, and their response was generally scored as 3 or 4. On the other hand, the AVR 122 line with *Pm7* showed a resistance response to all isolates and was therefore scored as 1. The cultivar ‘Canyon’, which also carried the *Pm7* gene, showed a different infection profile compared to APR 122. It was resistant to three isolates, intermediate to three other isolates, and susceptible to four isolates. The cultivar ‘Rollo’ had two resistance genes *Pm3+8*, but it showed a susceptible response to six isolates and an intermediate and resistant reaction to four isolates. The reference line AVE2406 with the *Pm9* gene showed an intermediate response against the set of isolates, which was mostly scored as 2. The lines AVE2925 (*Pm10*) and CN113536 (*Pm11*) exhibited an intermediate and susceptible response to isolates. The level of infection was scored as 2, 3, and 4. The *A. sterilis* CN67383 genotype carrying the *Pm12* gene was resistant to many isolates, but it also showed susceptible and intermediate responses to some of them. The *A. strigosa* genotype (Pl51586) had been identified as an effective source of resistance, but with an uncharacterized gene, and presented a resistant response to most isolates (Table 1).

The conducted host–pathogen tests indicated that oat cultivars originating from Central Europe had a low level of resistance to powdery mildew (Table 2). All of the cultivars from Poland showed complete susceptibility to the *B. graminis* f. sp. *avenae* isolates used in the host–pathogen tests. Out of 19 cultivars from the Czech Republic, 18 were found to be susceptible to all *B. graminis* f. sp. *avenae* isolates tested. However, the cultivar ‘Merlin’ showed a different infection profile compared to other cultivars. It was resistant to three *B. graminis* f. sp. *avenae* isolates, while it exhibited an intermediate response to an additional six isolates. Only one isolate presented a high level of virulence against this cultivar. Among the 13 cultivars from Germany, 9 were completely susceptible to powdery mildew isolates utilized in the host–pathogen tests in the present study. The cultivars ‘Bison’ and ‘Harmony’ were noted for their high level of resistance to all isolates used. Their resistance was rated as 0 and 1 on the Mains scale. The cultivars ‘Delfin’ and ‘Youkon’ also demonstrated high resistance to the *B. graminis* f. sp. *avenae* isolates investigated in the trial. Their resistance was scored as 0 and 1 against 9 out of 10 isolates tested. Concerning single isolates, they showed an intermediate response assessed as 2.

After analyzing the infection profiles of the cultivars and control genotypes, we inferred the presence of resistance genes in the study cultivars. The cultivars ‘Bison’ and ‘Harmony’ exhibited an infection profile that closely matched the infection profile of the APR 122 line with the *Pm7* gene, indicating the presence of this gene in these cultivars. Similarly, the infection profiles of the cultivars ‘Delfin’ and ‘Youkon’ were also similar to the APR 122 line carrying the *Pm7* gene. The infection profile of the Czech cultivar ‘Merlin’ was similar to those of the control forms with the *Pm7* (APR122) and *Pm9* (AVE2406) genes, but it was not sufficient to identify the genes that caused resistance in this cultivar. The resistance response of this cultivar may be a result of the interaction of resistance genes or a completely different source that was not included in the control set. These results indicated that the level of this resistance was adequate to defend the cultivar against pathogen attack and disease development.

## 3. Discussion

Based on the postulates of integrated plant management, one of the methods of controlling pathogens is the breeding of resistant cultivars. Before introducing cultivars with genetically determined resistance into breeding, it is important to take two important steps. Firstly, a thorough description of the pathogen population should be provided. Secondly, the level of resistance of the currently cultivated cultivars in a given area should be characterized [16,31]. Powdery mildew is one of the most dangerous fungal diseases occurring in oats [9,10]. Changes in the pathogen population and the emergence of disease outbreaks in various regions of the world mean that the attention of breeders should be focused on introducing cultivars with resistance genes effective against this pathogen [32].

According to reports from the literature, the *Pm1*, *Pm3*, *Pm6*, and *Pm7* genes have so far been identified in oat cultivars. Hsam et al. [23,24] and Hsam and Zeller [25] investigated the resistance of oat cultivars and breeding lines. They found that only a few showed resistance corresponding to the *Pm6*, *Pm3*, and *Pm1* genes. Okoń [27] and Okoń et al. [26] investigated the resistance of Polish oat cultivars and found that most of them were susceptible. They identify only *Pm3*, *Pm1*, and *Pm6* genes in a few cultivars. These studies showed that the *Pm1*, *Pm6*, and *Pm3* genes were used in oat breeding programs, but the number of varieties possessing these genes was not very large. Research conducted by Okoń [32] showed that the resistance conditioned by this gene has already been overcome by existing breeds of pathogens. These genes are no longer effective in Poland; however, the *Pm6* gene is still effective against the pathogen population in Ireland, the *Pm1* gene is effective in the Czech Republic, and the *Pm3* gene has maintained a high level of resistance in Ireland and Finland [33]. In recent years, the presence of the *Pm7* gene in German varieties has been confirmed [21]. Many previous studies have shown that *Pm7* was highly resistant in both seedling and adult plant stages [23,24,32,34]. The *Pm2*, *Pm4*, and *Pm5* genes have not been identified among the oat varieties analyzed so far [23,24,25,26,27]. These genes are currently most effective against powdery mildew [33,35]. There is no information in the available literature regarding the level of resistance of varieties cultivated in recent years. In this study, we conducted a resistance test on 46 oat cultivars from Poland, Germany, and the Czech Republic. Out of these cultivars, only five displayed resistance to the *B. graminis* f. sp. *avenae* isolates used in the test. Among these cultivars, we identified the *Pm7* gene in four of them. The infection profiles of these cultivars matched the infection profile of the APR122 line. However, in one cultivar, we were unable to determine the presence of the resistance gene through the analysis of the infection profile. Even with further studies using a larger number of isolates, we were unable to specify which resistance genes, of those described so far, are present in the Merlin variety. Based on our research, it appears that resistance to powdery mildew is not a widely available trait in oat breeding programs. We found that only one gene was identified in the tested cultivars. Relying on only one source of resistance in cultivars can create strong pressure on the pathogen population, leading to a relatively quick breaking of this resistance. Moreover, using the same set of resistance genes in breeding practices frequently results in the selection of pathogen pathotypes with corresponding virulence genes, leading to a breakdown of gene-conditioned resistance [36,37]. Our findings suggest that the resistance conditioned by the *Pm7* gene may be weakening and could potentially be broken by the *B. graminis* f. sp. *avenae* pathotypes in the future. A similar situation occurred in the case of the *Pm1*, *Pm3*, and *Pm6* genes, which were present for many years in varieties from different countries. Currently, these genes should not be used in breeding programs due to their poor efficiency.

Our analysis revealed that the oat cultivars currently grown in Central Europe have low levels of resistance to powdery mildew. Only a few of them possess one effective resistance gene. Due to the observed climate changes and the rapid adaptation of pathogens to new environmental conditions, it is crucial to introduce a wider pool of genes that determine the pathogen resistance of cultivars [38,39]. This will help protect these cultivars against new virulent pathotypes that may emerge in the future. To obtain long-term resistance, it is important to introduce several single effective genes or build gene pyramids that would allow for the long-term protection of plants against pathogen attack [40]. The literature provides numerous examples of effective sources of resistance that can be utilized to increase oat resistance, both in the form of single genes and well-selected gene pyramids [41,42,43,44,45,46].

## 4. Materials and Methods

The plant material used for the study comprised 46 present-day cultivars of common oats, with 14 being from Poland, 19 from the Czech Republic, and 13 from Germany. These cultivars are commonly used by farmers and were collected from native breeders in each country (COBORU, (Poznan, Poland), Saaten-Union, (Isernhagen, Germany) and Selgen (Sibřina, Czech Republic)).

A set of cultivars and lines with known resistance genes (*Pm*) were used as the control: Jumbo (*Pm1*), Mostyn (*Pm3*), AV1860 (*Pm4*), Am25 (*Pm5*), Bruno (*Pm6*), APR122 (*Pm7*), Rollo (*Pm3+Pm8*), AVE2406 (*Pm9*), AVE2925 (*Pm10*), CN113536 (*Pm11*), and CN 67383 (*Pm12*). Okoń et al. [35] showed that resistance conditioned by *Pm7* in the cultivar Canyon and line APR122 was different, so we decided to add this cultivar as a control in the presented study. In addition, the Pl51586 (*U _A. strigosa_*) genotype was identified as an effective source of resistance in our previous study [47] and was also included in the present work. The cultivar Fuchs was used as a susceptible control (Table 1).

The level of resistance of the analyzed cultivars was determined based on the infection profile of 10 single-spore *B. graminis* f. sp. *avenae*. The isolates were obtained from populations sampled in different parts of Poland in different years. Moreover, isolates were characterized by different infection patterns to the control genotypes.

The host–pathogen tests were carried out on the first leaves of 10-day-old seedlings of oat genotypes according to a modified method described by [23,24]. Leaf fragments were put on round culture plates half-filled with agar (6 g of agar per 1 L of water and 35 mg × 11 of benzimidazole). Plates with leaf fragments were inoculated using an inoculation tower by placing approx. 500–700 powdery mildew spores per 1 cm^2^. The plates were then incubated under appropriate conditions at approx. 17 °C and lighting intensity of approx. 4 kLx.

**Figure 1:**
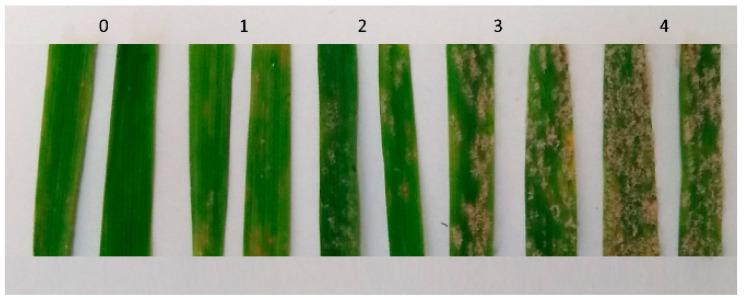
Photo of leaf fragments showing the different types of plant response to *B. graminis* f. sp. *avenae* infection.

The level of infection of the tested cultivars was determined ten days after infection with the isolates using the modified Mains scale [30], where 0 = no visible symptoms; 1 = very resistant, single colonies; 2 = intermediate resistance, moderate mycelium sporulating; 3 = moderately susceptible, extensive mycelium, more sporulation; and 4 = highly susceptible, large colonies, and abundant sporulation (Figure 1). To confirm the response of the tested cultivars to the used *B. graminis* f. sp *avenae* isolates, all tests were performed in three replications. In the cases when the genotype response to the applied isolate in the replications was different, as a result, the highest score was taken.

## Figures and Tables

**Table 1:** Standard differential set of oat lines and cultivars with described resistant genes.

Cultivar/Line	Gene Symbol	*Blumeria graminis* f. sp. *avenae* Isolates									
		PL 4/2018	STR 1/2029	F 4/2018	B 2014	F 2/2019	D 22019	Z 2015	CHR 4/2018	MH4	CZ 3/2019
Jumbo	*Pm1*	4	4	3	0	4	4	4	4	4	4
Cc37722	*Pm2*	0	0	0	0	0	0	0	0	0	0
Mostyn	*Pm3*	0	4	2	4	4	0	4	4	4	4
AV1860	*Pm4*	0	0	0	0	0	0	0	0	0	0
Am25	*Pm5*	0	0	0	0	0	0	0	0	0	0
Bruno	*Pm6*	4	4	3	4	4	4	4	4	4	4
APR 122	*Pm7*	1	1	1	1	1	1	0	1	0	0
Canyon	*Pm7*	2	1	1	4	2	1	4	3	4	2
Rollo	*Pm3+8*	1	2	3	2	3	0	4	4	4	4
AVE2406	*Pm9*	2	1	1	2	1	2	2	1	2	0
AVE2925	*Pm10*	4	2	1	4	2	2	1	4	3	1
CN113536	*Pm11*	4	3	2	3	2	4	2	4	3	3
CN67383	*Pm12*	0	3	0	0	0	0	2	0	0	2
Pl51586	*U _A. strigosa_*	0	0	2	0	1	1	0	4	0	0
Fuchs	-	4	4	3	4	4	0	4	4	4	4

**Table 2:** Results of infection of oat cultivars from Central Europe with 10 isolates *Blumeria graminis* f. sp *avenae* using the Mains scale (0–4) [30].

	Culitivar	Origin	PL 4/2018	STR 1/2029	F 4/2018	B 2014	F 2/2019	D 22019	Z 2015	CHR 4/2018	MH4	CZ 3/2019
1	Agent	POL	4	4	4	4	4	4	4	4	4	4
2	Arden	POL	4	4	4	4	4	4	4	4	4	4
3	Berdysz	POL	4	4	4	4	4	4	4	4	4	4
4	Bingo	POL	4	4	4	4	4	4	4	4	4	4
5	Breton	POL	4	4	4	4	4	4	4	4	4	4
6	Elegant	POL	4	4	4	4	4	4	4	4	4	4
7	Figaro	POL	4	4	4	4	4	4	4	4	4	4
8	Harnaś	POL	4	4	4	4	4	4	4	4	4	4
9	Komfort	POL	4	4	4	4	4	4	4	4	4	4
10	Krezus	POL	4	4	4	4	4	4	4	4	4	4
11	Nawigaror	POL	4	4	4	4	4	4	4	4	4	4
12	Paskal	POL	4	4	4	4	4	4	4	4	4	4
13	Romulus	POL	4	4	4	4	4	4	4	4	4	4
14	Zuch	POL	4	4	4	4	4	4	4	4	4	4
15	Abel	CZECH	4	4	4	4	4	4	4	4	4	4
16	Atego	CZECH	4	4	4	4	4	4	4	4	4	4
17	Azur	CZECH	4	4	4	4	4	4	4	4	4	4
18	Caroline	CZECH	4	4	4	4	4	4	4	4	4	4
19	Gregor	CZECH	4	4	4	4	4	4	4	4	4	4
20	Kertag	CZECH	4	4	4	4	4	4	4	4	4	4
21	Korok	CZECH	4	4	4	4	4	4	4	4	4	4
22	Neklan	CZECH	4	4	4	4	4	4	4	4	4	4
23	Norbert	CZECH	4	4	4	4	4	4	4	4	4	4
24	Obelisk	CZECH	4	4	4	4	4	4	4	4	4	4
25	Oberon	CZECH	4	4	4	4	4	4	4	4	4	4
26	Raven	CZECH	4	4	4	4	4	4	4	4	4	4
27	Rozmar	CZECH	4	4	4	4	4	4	4	4	4	4
28	Sagar	CZECH	4	4	4	4	4	4	4	4	4	4
29	Vok	CZECH	4	4	4	4	4	4	4	4	4	4
30	Kamil	CZECH	4	4	4	4	4	4	4	4	4	4
31	Patrik	CZECH	4	4	4	4	4	4	4	4	4	4
32	Celeste	CZECH	4	4	4	4	4	4	4	4	4	4
33	Merlin	CZECH	1	2	2	4	2	2	1	2	2	1
34	Delfin	GER	0	1	2	0	0	1	0	1	1	0
35	Bison	GER	0	1	0	1	0	0	1	0	0	1
36	Harmony	GER	0	0	0	0	0	1	0	0	0	0
37	Youkon	GER	2	1	1	1	1	0	1	0	0	1
38	Lion	GER	4	4	4	4	4	4	4	4	4	4
39	Monsun	GER	4	4	4	4	4	4	4	4	4	4
40	Perun	GER	4	4	4	4	4	4	4	4	4	4
41	Poseidon	GER	4	4	4	4	4	4	4	4	4	4
42	Scorpion	GER	4	4	4	4	4	4	4	4	4	4
43	Symphony	GER	4	4	4	4	4	4	4	4	4	4
44	Ivory	GER	4	4	4	4	4	4	4	4	4	4
45	Apollon	GER	4	4	4	4	4	4	4	4	4	4
46	Moby	GER	4	4	4	4	4	4	4	4	4	4

## Data Availability

The data is contained within the manuscript.
